# Efficacy and Safety of L-Carnitine Treatment for Chronic Heart Failure: A Meta-Analysis of Randomized Controlled Trials

**DOI:** 10.1155/2017/6274854

**Published:** 2017-04-13

**Authors:** Xiaolong Song, Huiyan Qu, Zongguo Yang, Jingfeng Rong, Wan Cai, Hua Zhou

**Affiliations:** ^1^Department of Cardiology, Shuguang Hospital, Shanghai University of Traditional Chinese Medicine, Shanghai 201203, China; ^2^Department of Cardiology, Yancheng Traditional Chinese Medicine Hospital Affiliated to Nanjing University of Chinese Medicine, Yancheng 224001, China; ^3^Department of Traditional Chinese Medicine, Shanghai Public Health Clinical Center, Fudan University, Shanghai 201508, China

## Abstract

*Background*. Whether additional benefit can be achieved with the use of L-carnitine (L-C) in patients with chronic heart failure (CHF) remains controversial. We therefore performed a meta-analysis of randomized controlled trials (RCTs) to evaluate the effects of L-C treatment in CHF patients.* Methods*. Pubmed, Ovid Embase, Web of Science, and Cochrane Library databases, Chinese National Knowledge Infrastructure (CNKI) database, Wanfang database, Chinese Biomedical (CBM) database, and Chinese Science and Technology Periodicals database (VIP) until September 30, 2016, were identified. Studies that met the inclusion criteria were systematically evaluated by two reviewers independently.* Results*. 17 RCTs with 1625 CHF patients were included in this analysis. L-C treatment in CHF was associated with considerable improvement in overall efficacy (OR = 3.47, *P* < 0.01), left ventricular ejection fraction (LVEF) (WMD: 4.14%, *P* = 0.01), strike volume (SV) (WMD: 8.21 ml, *P* = 0.01), cardiac output (CO) (WMD: 0.88 L/min, *P* < 0.01), and E/A (WMD: 0.23, *P* < 0.01). Moreover, treatment with L-C also resulted in significant decrease in serum levels of BNP (WMD: −124.60 pg/ml, *P* = 0.01), serum levels of NT-proBNP (WMD: −510.36 pg/ml, *P* < 0.01), LVESD (WMD: −4.06 mm, *P* < 0.01), LVEDD (WMD: −4.79 mm, *P* < 0.01), and LVESV (WMD: −20.16 ml, 95% CI: −35.65 to −4.67, *P* < 0.01). However, there were no significant differences in all-cause mortality, 6-minute walk, and adverse events between L-C and control groups.* Conclusions*. L-C treatment is effective for CHF patients in improving clinical symptoms and cardiac functions, decreasing serum levels of BNP and NT-proBNP. And it has a good tolerance.

## 1. Introduction

Chronic heart failure (CHF) is a complex clinical syndrome characterized by decreased myocardial contractility, hemodynamic abnormality, and neuroendocrine activation. It is a global public health problem affecting estimated 26 million worldwide [1]. Currently, the neurohormonal antagonists (ACE-inhibitors, beta-blockers, angiotensin receptor blockers, and mineralocorticoid receptor antagonists) are recommended for CHF as cornerstones [2, 3]. However, it remains a leading cause of morbidity and mortality throughout the world.

Recently, there has been a growing appreciation of the complex metabolic processes underlying HF pathophysiology and symptoms [4]. In fact, the failing heart may be defined as “an engine out of fuel” [5]. L-carnitine is a vitamin-like and modified amino acid that plays an important role in supporting the body's metabolic activities. There is growing evidence that high concentrations of L-C provide beneficial effects in various diseases such as coronary artery disease, congestive heart failure, peripheral vascular diseases, type 2 diabetes, dyslipidemia, and hypertension [6].

However, the clinical guidelines about nutritional supplements in different countries are not consistent. In Chinese guideline, nutritional supplements (trimetazidine, coenzyme Q10, and L-C) may be helpful to CHF [7]. But, as a treatment of heart failure, L-C has not been recommended in patients with current or prior symptoms of heart failure with reduced ejection fraction and heart failure with preserved ejection fraction in the American guideline [3]. The recommendation of nutritional supplements has not been proposed in the European guideline [2].

In addition, two meta-analyses of RCTs has been performed to assess the therapeutic effects of L-C in the secondary prevention of cardiovascular disease [8, 9], but there is no meta-analysis of RCTs in CHF. Over the past few decades, several small RCTs have been conducted to evaluate the effects of L-C treatment in patients with CHF. Thus, we performed a meta-analysis of RCTs with critical inclusion and exclusion criteria to evaluate the efficacy and tolerance of L-C.

## 2. Materials and Methods

### 2.1. Search Strategy

We searched Pubmed, Ovid Embase, Web of Science, and Cochrane Library databases, Chinese National Knowledge Infrastructure (CNKI) database, Wanfang database, Chinese Biomedical (CBM) database, Chinese Science and Technology Periodicals database (VIP) until September 30, 2016. The following medical subject headings were used: “L-carnitine,” “carnitine,” “levocarnitine,” “novain,” “L-cthernitine,” “Vitamin BT,” “Bicarnesine,” “heart failure,” “cardiac dysfunction,” “cardiac insufficiency,” “cardiac inadequacy,” “cardiomyopathy,” and “ventricular dysfunction.” Electronic searches were supplemented with manual searches of reference lists of all retrieved review articles, primary studies, and abstracts from meetings to identify other studies not found in the electronic searches. Literature was searched by two authors (X. Song and Z. Yang) independently. The search was limited to human subjects, with no restriction for language.

### 2.2. Study Selection

Two authors independently selected trials and discussed with each other when inconsistencies were found. Articles that meet the following criteria were included: (1) study types, randomized controlled trials; (2) participants, chronic heart failure patients (age ≥ 18 years); (3) interventions, L-C with placebo, routine, or conventional treatment; (4) outcome measures, studies that used one or more of the following measurements were eligible: all-cause mortality, cardiovascular events, New York Heart Association (NYHA) classification, overall efficacy, exercise capacity (i.e., 6-minute walk), changes in cardiac function parameters (i.e., left ventricular ejection fraction (LVEF), strike volume (SV), cardiac output (CO), E/A, left ventricular end-systolic dimension (LVESD), left ventricular end-diastolic dimension (LVEDD), and left ventricular end-systolic volume (LVESV)), brain natriuretic peptide (BNP), N-terminal pro-brain natriuretic peptide (NT-proBNP), and adverse events. (5) Full texts available. Studies without randomized method from CNKI, CBM, and VIP were excluded. Studies that included other nutritional supplements (i.e., trimetazidine, coenzyme Q10) were excluded. Nonrandomized evaluations, pharmacokinetic studies, animal/laboratory studies, and general reviews were excluded, and duplicated publications reporting the same groups of patients were also excluded (Figure S1 in Supplementary Material available online at https://doi.org/10.1155/2017/6274854).

### 2.3. Quality Assessment

The methodological qualities of the included RCTs were assessed according to Cochrane Collaboration's tool described in Handbook version 5.1.0 [10]. Two authors (X. Song and Z. Yang) assessed the quality independently, and inconsistency was discussed with a third review author (H. Zhou) who acted as an arbiter.

### 2.4. Data Extraction

Two researchers read the full texts independently and extracted the following contents: publication data (first author's name, year of publication), study characteristic (study design, sample size, follow-up duration, inclusion criteria, and endpoints), patient characteristics (age, gender, NYHA classification, cardiac histology, and LVEF), treatment protocol (L-C dose), and outcome measures (all-cause mortality, overall efficacy, NYHA classification, 6-minute walk, LVEF, SV, CO, E/A, LVEDD, LVESD, LVESV, BNP, NT-proBNP, and adverse events). Authors were contacted by e-mail for additional information if data were unavailable.

### 2.5. Statistical Methods

Data were processed in accordance with the Cochrane Handbook [10]. Intervention effects were expressed with odds ratios (ORs) and associated 95% confidence intervals (CIs) for dichotomous data. By contrast, the effects were expressed with mean differences and 95% CIs for continuous data. Statistical heterogeneity was measured using *I*^2^ statistic and *I*^2^ statistic with significance set at *I*^2^ greater than 50% [11].

The fixed-effects model was first used for meta-analyses. The random-effects model was used in the presence of heterogeneity. Description analysis was performed when the quantitative data could not be pooled. Intention-to-treat (ITT) principle was used. Review Manage (v.5.1; the Cochrane Collaboration) was used for data analysis.

## 3. Results

### 3.1. Study and Patient Characteristics

The flow of selecting studies for the meta-analysis is shown in Figure S1. Briefly, among the initial 2870 reports, 468 articles were retrieved for detailed evaluation, and 17 RCTs [12–28] enrolling 1625 participants that fulfilled the inclusion criteria were finally analyzed. The study and patient characteristics are shown in Table 1. L-C dosage ranged from 1.5 to 6 g/day and follow-up periods from 7 days to 3 years.

### 3.2. Methodological Quality Assessment

**Figure 1:**
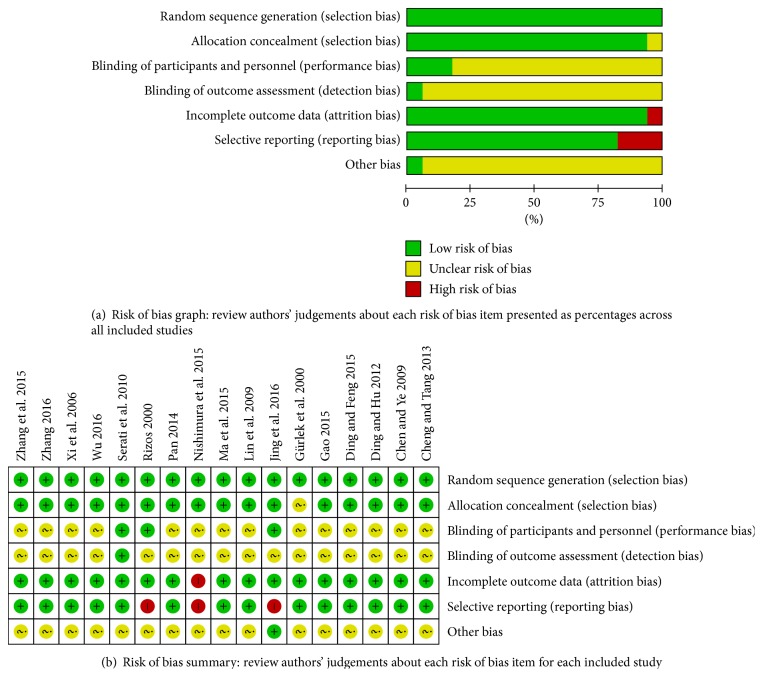
Risk of bias assessment.

**Figure 2:**
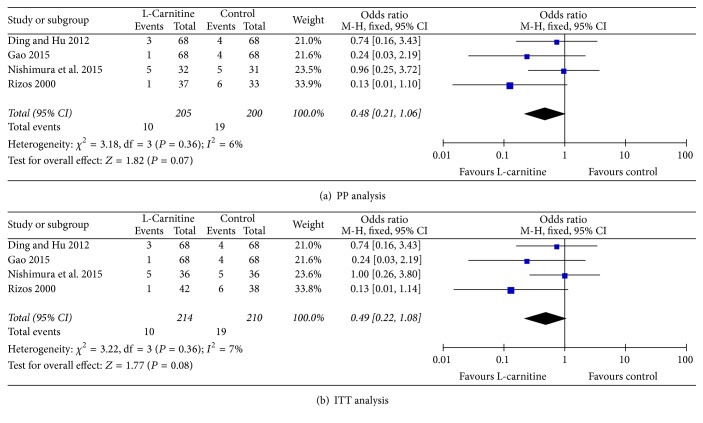
Forest plots for all-cause mortality.

All studies included in this meta-analysis were randomized controlled trials. The quality assessment of the included RCTs is shown in Figure 1. Four studies [12, 13, 21, 26] did not report the method of randomization, whereas the others reported a randomization number sequence or adaptive minimization randomization scheme. There were three studies [13, 17, 26] using the blind method; blind methods of all the other studies are unclear. One study [21] had high performance bias for the reason that more than 20% of patients were lost to follow-up. Selective reporting was found in three studies [13, 21, 26] because they did not present the ITT analysis data. These studies had high performance bias and detection bias. The other potential biases were unclear in these trials (Figure 2). Because most of the studies were conducted in China, we cautiously drew the conclusion that publication bias might have been present in the meta-analysis. The differences of treatment period would influence the outcomes of chronic heart failure patients. Eight studies [14, 18–20, 22, 24, 27, 28] had a treatment period of 2 weeks, and the follow-up period of other studies still ranged variously. In this condition, we conducted a subgroup analysis in these studies with 2 -week treatment period (Figure S3).

### 3.3. All-Cause Mortality for Cardiac Causes

Four studies [13, 18, 21, 23] reported all-cause mortality. As shown in Figure 2, no significant differences were found in heterogeneity in both per-protocol (PP) and ITT analysis (*I*^2^ = 6%, *P* = 0.36 and *I*^2^ = 7%, *P* = 0.36, resp.). The results of PP analysis showed that all-cause mortality in the L-C group was not lower than control (OR = 0.48, 95% CI: 0.21 to 1.06, *P* = 0.07, Figure 2(a)). ITT analysis also showed no differences in all-cause mortality between heart failure patients in both groups (OR = 0.49, 95% CI: 0.22 to 1.08, *P* = 0.08, Figure 2(b)).

### 3.4. Functional Capacity

**Figure 3:**
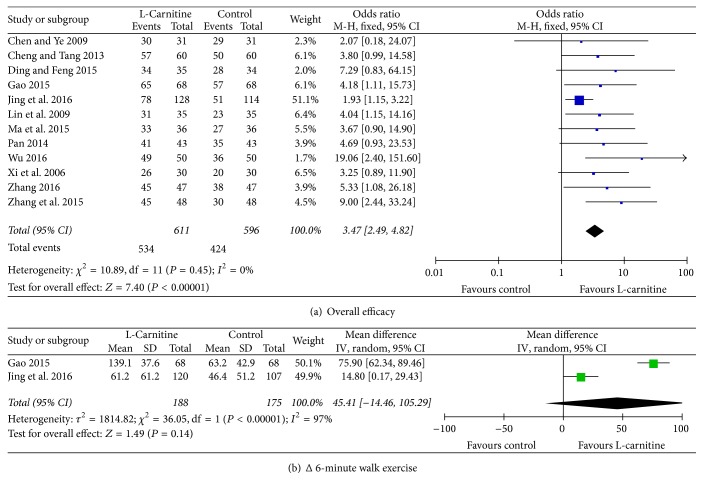
Forest plots for functional capacity.

The improvement in cardiac function (decreased NYHA class) was rated as overall efficacy. The endpoint was a decrease of at least one NYHA class, with efficacy rated as effective (decrease of two classes, decrease of one class) or ineffective (no class change). The overall efficacy consisted of excellent and effective rate. Twelve studies [14–16, 19, 20, 22–28] reported overall efficacy; there were no significant differences in heterogeneity (*I*^2^ = 0%, *P* = 0.45). Meta-analysis indicated that patients who received L-C treatment had higher overall efficacy than those in control group (OR = 3.47, 95% CI: 2.49 to 4.82, *P* < 0.01, Figure 3(a)). Subgroup analysis of 2-week treatment period also demonstrated that overall efficacy of L-C treatment was higher than the control group (OR = 5.11, 95% CI: 2.87 to 9.10, *P* < 0.01, Figure S3). Considering significant heterogeneity between studies [23, 26] when we compared difference values about Δ6-minute walk, meta-analysis with random-effect model revealed that there were no significant differences in improvement of exercise tolerance between the two treatments (WMD: 45.41 m, 95% CI: −14.46 to 105.29, *P* = 0.14, Figure 3(b)).

### 3.5. Serum Markers

**Figure 4:**
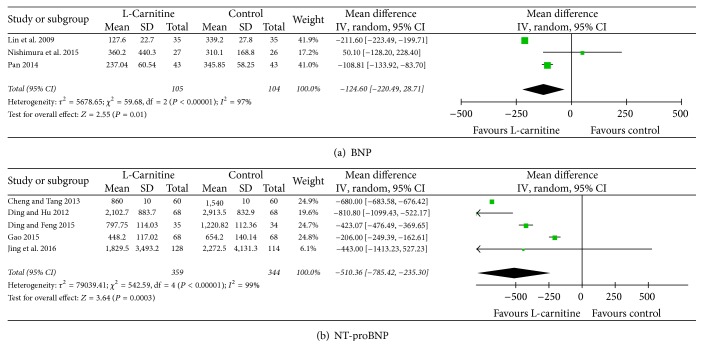
Forest plots for serum markers.

Heterogeneity was significant among the included studies [16, 18–23, 26] in which there were changes in serum levels of BNP and NT-proBNP (*I*^2^ = 97%, *P* < 0.01 and *I*^2^ = 99%, *P* < 0.01, resp.). Thus, a random-effects model was applied; we found that in serum levels of BNP and NT-proBNP were significantly decreased in the L-C group compared with those in the control group (WMD: −124.60 pg/ml, 95% CI: −220.49 to −28.71, *P* = 0.01; WMD: −510.36 pg/ml, 95% CI: −785.42 to −235.30, *P* < 0.01, resp., Figure 4). Subgroup analysis indicated that levels of NT-proBNP were significantly decreased in the L-C group (WMD: −612.44 pg/ml, 95% CI: −829.41 to −395.47, *P* < 0.01, Figure S3).

### 3.6. Left Ventricular Structure and Function

**Figure 5:**
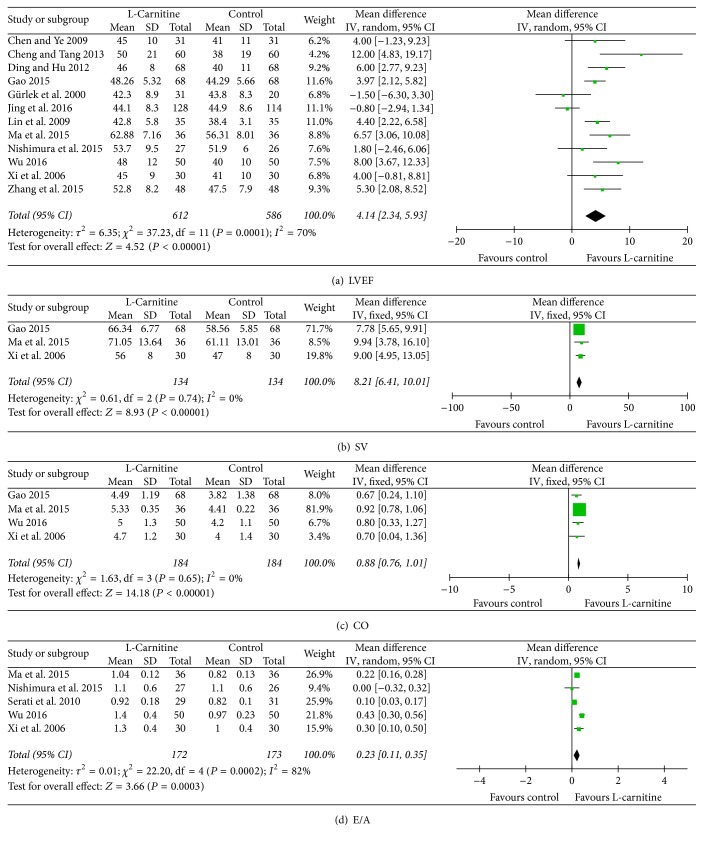
Forest plots for left ventricular structure and function (I).

**Figure 6:**
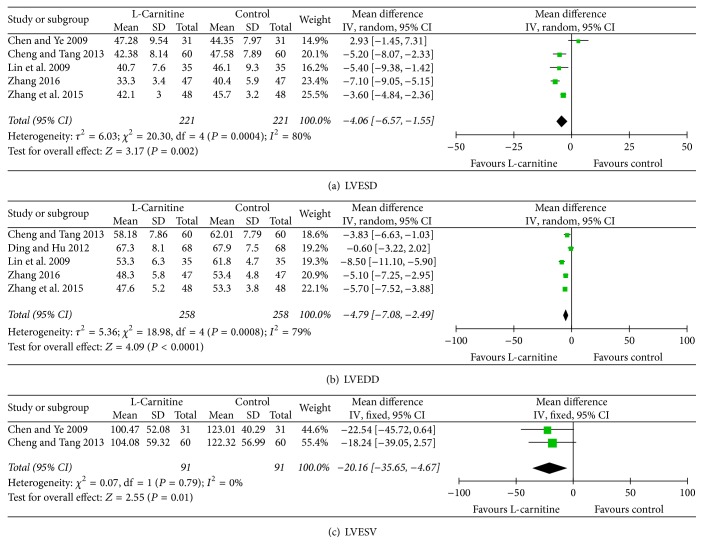
Forest plots for left ventricular structure and function (II).

Twelve studies [12, 14–16, 18, 19, 21, 23–27] provided data on LVEF, considering that significant heterogeneity was found among the included studies (*I*^2^ = 70%, *P* < 0.01); we used a random-effect model and a profound improvement in LVEF was observed in patients who received L-C therapy (WMD: 4.14%, 95% CI: 2.34 to 5.93, *P* = 0.01, Figure 5(a)). As shown in Figures 5(b) and 5(c), no significant differences were found in heterogeneity in both SV [14, 23, 24] and CO [14, 23, 24, 27] analysis (*I*^2^ = 0%, *P* = 0.74 and *I*^2^ = 0%, *P* = 0.65, resp.). SV and CO were significantly higher in patients who received L-C therapy than control group (WMD: 8.21 ml, 95% CI: 6.41 to 10.01, *P* = 0.01; WMD: 0.88 L/min, 95% CI: 0.76 to 1.01, *P* < 0.01, resp., Figures 5(b) and 5(c)). Heterogeneity was significant among the studies [14, 17, 21, 24, 27] when comparing E/A (*I*^2^ = 82%, *P* < 0.01). Thus, a random-effects model was used. Our data revealed that E/A was significantly higher for patients who received L-C treatment (WMD: 0.23, 95% CI: 0.11 to 0.35, *P* < 0.01, Figure 5(d)). In addition, our results indicated that L-C was associated with a significant drop in LVESD [15, 16, 19, 25, 28] and LVEDD [16, 18, 19, 25, 28] for patients (WMD: −4.06 mm, 95% CI: −6.57 to −1.55, *P* < 0.01; WMD: −4.79 mm, 95% CI: −7.08 to −2.49, *P* < 0.01, resp., Figures 6(a) and 6(b)). We also found that LVESV [15, 19] was significantly decreased in response to L-C therapy (WMD: −20.16 ml, 95% CI: −35.65 to −4.67, *P* < 0.01, Figure 6(c)). According to subgroup analysis of 2-week treatment period, statistically significant association was found between LVEF, SV, CO, E/A, LVESD, and LVEDD (WMD: 6.63%, 95% CI: 4.78 to 8.47, *P* < 0.01; WMD: 9.28 ml, 95% CI: 5.90 to 12.67, *P* < 0.01; WMD: 0.90 L/min, 95% CI: 0.78 to 1.03, *P* < 0.01; WMD: 0.31, 95% CI: 0.16 to 0.46, *P* < 0.01; WMD: −6.50 mm, 95% CI: −8.11 to −4.89, *P* < 0.01; WMD: −3.24 mm, 95% CI: −5.93 to −0.55, *P* < 0.01, resp., Figure S3).

### 3.7. Major Adverse Events

Six studies [12, 14, 16, 24, 25, 28] reported that there were no adverse events related to L-C. Four reports [13, 20, 23, 26] contained data on specific adverse events. Dry mouth and gastrointestinal problems were the major adverse events, and rash was reported by a study of Pan [20] in both L-C group (4/43) and control group (2/43). No participants have withdrawn from the study for the reason of adverse events. Meta-analysis demonstrated no differences in adverse events between patients in both groups (5.4% versus 5.8%, OR = 0.92, 95% CI: 0.44 to 1.92, *P* = 0.83, Figure S2). The other seven trials did not report adverse events.

## 4. Discussion

L. H. Opie indicated that “the heart is more than a pump. It is also an organ that needs energy from metabolism. A metabolic disease, ischaemia, should ideally be treated by metabolic therapy” [29]. CHF is currently conceived as a systemic and multiorgan syndrome with metabolic failure as basic mechanism. In fact, the failing heart may be defined as “an engine out of fuel” [5]. L-C is a natural constituent of human cells and participates in fatty acid metabolism. L-C plays an important role in lipid metabolism by acting as an obligatory cofactor for oxidation of fatty acids and facilitating the transport of long-chain fatty acids (LCFAs) across the mitochondrial membrane to provide enough adenosine triphosphate (ATP) for myocardial cells [30].

Although heart failure is not listed as one of indications in package insert of L-C, clinical application of L-C has shown significant relief of heart failure, which was confirmed in our research.

Recently, DiNicolantonio et al. (2013) [9] conducted a meta-analysis of L-C in the secondary prevention of cardiovascular disease. Compared with placebo, L-C was associated with a 27% reduction in all-cause mortality, a 65% reduction in ventricular arrhythmias, and a 40% reduction in angina in patients experiencing an acute myocardial infarction (AMI). However, our study suggests that the additional use of L-C failed to reduce all-cause mortality in CHF patients.

Chronic oral L-C supplementation has been shown to ameliorate factors associated with metabolic syndrome and cardiovascular disease, such as arterial hypertension, cholesterol levels, impaired glucose tolerance, and insulin resistance. L-C appears to be particularly suitable as a treatment for metabolic syndrome patients, who are often obese, insulin resistant, and hypertensive [31–33]. One included study [27] suggested that L-C can significantly ameliorate total cholesterol, blood sugar, and cardiac function and improve clinical symptoms of patients for heart failure patients with diabetes mellitus.

Our findings of this meta-analysis are that the beneficial effects have been shown by the increase of overall efficacy, LVEF, strike volume, cardiac output, and E/A, by the decrease of left ventricular end-diastolic diameter, left ventricular end-systolic diameter, and left ventricular end-systolic volume and serum levels of BNP and NT-proBNP, and with satisfactory safety. Some reports [21, 24, 25] indicated that clinical symptom, cardiac function, and renal function in CHF patients with renal insufficiency were more likely to be ameliorated with L-C treatment.

According to the “energy starvation” hypothesis, which induces that insufficient ATP supply underlies the contractile dysfunction presenting in heart failure [34], it seems reasonable that L-C improves energy metabolism in cardiomyocytes, which may finally translate into mechanical efficiency and contribute to the improvement of clinical symptoms and cardiac function. Furthermore, noteworthy is that L-C exerts cardioprotective effects through alternative mechanisms, such as oxidative stress [35], nitric oxide [36], arterial hypertension, cardiac inflammation and fibrosis [37–39], and interstitial remodeling [40], as well as by improving endothelial function [41].

It is noteworthy that, based on existing conventional treatment, L-C was used in treatment of energy metabolism disorder. It acted as a supplementation to preexisting treatment rather than a replacement. Chronic heart failure is a disease that requires multitargeted and phase dependent therapeutic methods. Our research showed that L-C represents a safe and effective adjuvant therapy which, by increasing high energy phosphate for systolic and diastolic function, may have a synergistic effect with other drugs.

According to study, relatively high degree of heterogeneity was found regarding the following four indices: LVEF, BNP, NT-proBNP, and 6-minute walking distance. Through analyzing the included references, we speculated the following possible causes for the discrepancies: (1) different uses, doses of L-C utilized in these studies. (2) Different states of illness and stages of therapies of patients included in these studies. (3) Manufacturers and specifications of L-C being not clearly indicated in most of these studies.

Some limitations of our meta-analysis need to be acknowledged. Firstly, the methodological quality of included studies was less than optimal, so we were not able to exclude the potential risk of bias in these trials. Secondly, it is worth noticing that only 1625 patients were involved in the 17 RCTs, which justifies the performance of more large-scale RCTs for evaluating the impact of L-C treatment on CHF patients. Thirdly, the follow-up duration in these studies varied widely, from 7 days to 3 years. Owing to these limitations, our results are insufficient to recommend the method as a first-line treatment or to establish the quality of life and long-term results. Therefore, further research is required to more accurately assess the results of L-C for treating CHF.

In conclusion, our meta-analysis demonstrates that L-C treatment in CHF patients may improve clinical symptoms and cardiac function and decrease serum levels of BNP and NT-proBNP and has good tolerance.

## Supplementary Material

There were three main parts of the supplementary material. Figure S1 shows flow diagram of study selection, Figure S2 shows forest plots for adverse events, and Figure S3 shows forest plots for subgroup analysis of 2-week treatment period.

## Figures and Tables

**Table 1:** Baseline characteristics of studies included in this meta-analysis.

Study	Patient (L-C/control)	Age (mean, year) (L-C/control)	Male (*N*) (L-C/control)	L-C dose (g/day)	Follow-up duration	Ischemic cause (%)	NYHA class	LVEF (mean, %) (L-C/control)	Inclusion criteria	Endpoints
Gürlek 2000	51 (31/20)	64.3/66.2	27/17	2	1 month	100	3.21/3.32	37.8/41.5	Ischemic cardiomyopathy	LVEF, red cell superoxide dismutase activity, adverse events

Rizos 2000	80 (42/38)	50/48	19/20	2	Three years	0	III-IV	27/29	Dilated cardiomyopathy	Mortality, Weber classification, maximal time of cardiopulmonary exercise test, peak VO_2_ consumption, CO, adverse events

Xi 2006	60 (30/30)	63/63	18/16	3	14 days	42	III-IV	33/34	Chronic HF	Efficacy rated, SV, CO, CI, LVEF, E/A, NYHA class, adverse events

Chen 2009	62 (31/31)	68.5/70.8	20/22	3	10 days	35	III-IV	34/33	Chronic HF	Efficacy rated, NYHA class, LVEF, LVESD, LVESV

Lin 2009	70 (35/35)	43–78/42–76	18/19	3	20 weeks	20	III-IV	35.6/36.7	Chronic systolic HF, LVEF ≤ 40%	Efficacy rated, NYHA class, BNP, LVEF, LVEDD, LVESD, adverse events

Serati 2010	60 (29/31)	55/58	8/7	1.5	3 months	NA	II	NA	NYHA II, LVEF > 45%, mild diastolic dysfunction	Echocardiographic parameters (i.e., E, A, E′, E/A)

Ding 2012	136 (68/68)	75/74	41/40	2	14 days	NA	III-IV	37/38	Chronic congestive HF	Mortality, LVEDD, LVEF, NT-proBNP

Cheng 2013	120 (60/60)	57.9/70.1	34/36	3	15 days	31	III-IV	34/35	Chronic HF	Efficacy rated, NYHA class, LVEDD, LVESD, LVESV, LVEF, NT-proBNP, Scr, Cysc

Pan 2014	86 (43/43)	67.3/68.9	29/25	2	14 days	NA	NA	NA	Chronic HF	Efficacy rated, NYHA class, BNP, *β*-endorphin, adverse events

Nishimura, 2015	72 (36/36)	64.3/64.7	14/14	1	1 year	NA	IV	52/53	Chronic hemodialysis with HF needing hospitalization	Mortality, serum carnitine level, BNP, LVEF, E/A, LVMI, BMIPP

Ding 2015	69 (35/34)	64.2/64.5	25/23	3	14 days	100	II–IV	NA	Chronic HF, stable ischemic heart failure	Efficacy rated, NYHA class, NT-proBNP

Gao 2015	136 (68/68)	61–75/62–76	45/44	3	1 year	100	III-IV	35.43/36.10	Chronic HF, stable ischemic heart failure	Mortality, efficacy rated, NYHA class, NT-proBNP, 6-minute walk, LVEF, SV, CO, adverse events

Ma 2015	72 (36/36)	55.83/56.02	21/20	2	14 days	25	III-IV	44.12/43.74	Chronic HF, CRS	Efficacy rated, NYHA class, LVEF,SV,CO, E/A, Scr, BUN, Cysc, adverse events

Zhang 2015	96 (48/48)	45.9/47.2	33/34	3	7 days	58.3	III-IV	37.3/36.2	CRS without hemodialysis	Efficacy rated, NYHA class, LVEF, LVEDD, LVESD, Scr, BUN, adverse events

Jing 2016	261 (133/128)	51.9/52.4	84/70	6	7 days	NA	II–IV	41.12/40.39	Chronic HF, NYHA II-IV	Efficacy rated, NYHA class, LVEF, NT-proBNP, 6-minute walk, serum carnitine levels, adverse events

Wu 2016	100 (50/50)	74.22/73.70	26/28	2	15 days	NA	III-IV	35/31.10	Chronic HF with Diabetes	Efficacy rated, NYHA class, FPG, TC, LVEF, CO, E/A

Zhang 2016	94 (47/47)	56.3/58.3	27/29	3	15 days	NA	III-IV	NA	Chronic HF	Efficacy rated, NYHA class, LVEDD, LVESD, adverse events

L-C: L-carnitine; HF: heart failure; SV: strike volume; CO: cardiac output; CI: cardiac index; BNP: brain natriuretic peptide; NT-proBNP: N-terminal pro-brain natriuretic peptide; LVEF: left ventricle ejection fraction; mitral E: peak velocity of the early filling wave of the transmitral flow; mitral A: peak velocity of the atrial filling wave of the transmitral flow; E/A: mitral E/A; LVEDD: left ventricular end-diastolic dimension; LVESD: left ventricular end-systolic dimension; LVESV: left ventricular end-systolic volume; LVMI: Left ventricular mass index; NYHA: New York Heart Association; CRS: cardiorenal syndrome; BMIPP: I-*β*-methyliodophenyl pentadecanoic acid; FPG: fasting blood glucose; TC: total cholesterol; Scr: serum creatinine; BUN: blood urea nitrogen; Cysc: cystatin c; NA: not available.

## Abbreviations

L-C: L-Carnitine
CHF: Chronic heart failure
NYHA: New York Heart Association
BNP: Brain natriuretic peptide
NT-proBNP: N-terminal pro-brain natriuretic peptide
LVEF: Left ventricle ejection fraction
LVEDD: Left ventricular end-diastolic dimension
LVESD: Left ventricular end-systolic dimension
LVESV: Left ventricular end-systolic volume
mitral E: Peak velocity of the early filling wave of the transmitral flow
mitral A: Peak velocity of the atrial filling wave of the transmitral flow
E/A: Mitral E/A
CI: Confidence interval
WMD: Weighted mean differences.
