# Supplementary material for: Efficacy and Safety of L-Carnitine Treatment for Chronic Heart Failure: A Meta-Analysis of Randomized Controlled Trials
Source: Biomed Res Int. 2017 Apr 13;2017:6274854. doi: 10.1155/2017/6274854 (PMC5406747; doi:10.1155/2017/6274854)

**Figure S1** Flow diagram of study selection

**Included**

**Screening**

**Eligibility**

**Identification**

Studies included in quantitative synthesis (meta-analysis)
(n = 17)

Studies included in qualitative synthesis
(n = 17)

Full-text articles assessed for eligibility
(n = 468)

Records screened
(n = 468)

Full-text articles excluded, with reasons (n = 451):

**∙** Not RCT n =127

**∙** Animal experiment research n = 112

**∙** Chinese papers without randomized methods n = 197

∙ Data overlapping n =7

∙ Review n =6

∙ Patients received other therapies n =2

Records excluded
(n = 777)

Records after duplicates removed
(n = 1245)

Records identified through database searching
(n = 2870)

No additional records identified

through other sources

**Figure S2** Forest plots for adverse events


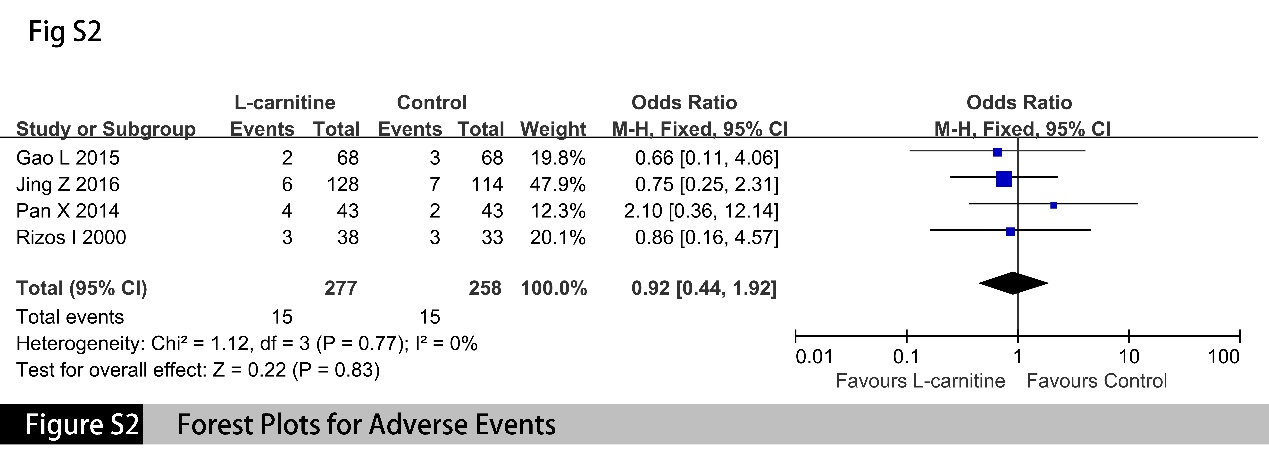


**Figure S3** Forest plots for subgroup analysis of 2 -weeks treatment period.


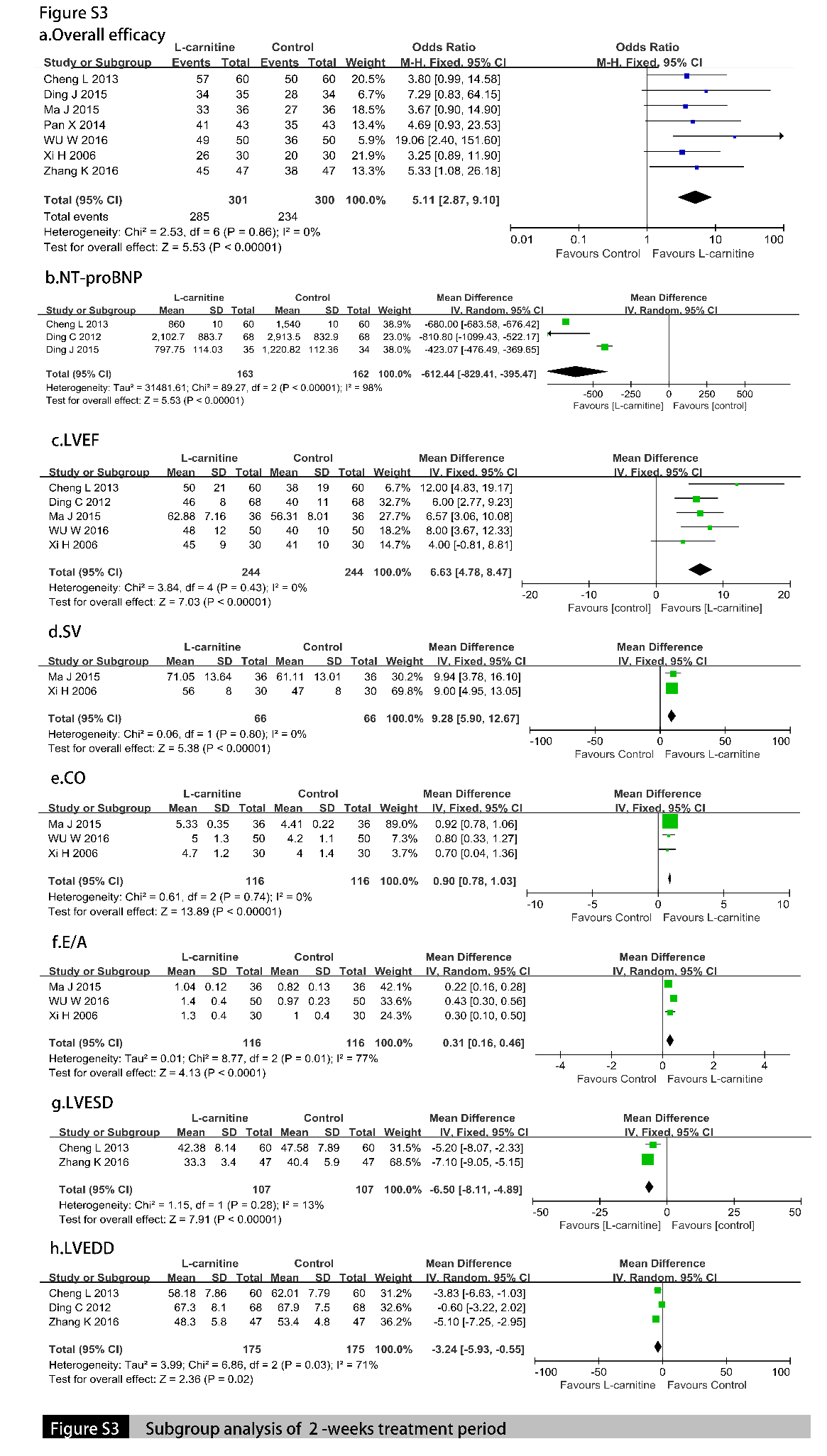

Supplement: Supplementary file 1 — There were three main parts of the supplementary material. Figure S1 shows flow diagram of study selection, Figure S2 shows forest plots for adverse events, and Figure S3 shows forest plots for subgroup analysis of 2-week treatment period. [file 6274854.f1.docx]
